# Barriers and facilitators to young children's physical activity and sedentary behaviour: a systematic review and synthesis of qualitative literature

**DOI:** 10.1111/obr.12562

**Published:** 2017-06-06

**Authors:** K. R. Hesketh, R. Lakshman, E. M. F. van Sluijs

**Affiliations:** ^1^ MRC Epidemiology Unit and Centre for Diet and Activity Research University of Cambridge Cambridge UK; ^2^ UCL Great Ormond Street Institute of Child Health London UK; ^3^ Public Health Directorate, Cambridgeshire County Council Cambridge UK

**Keywords:** Physical activity, preschool, qualitative, review

## Abstract

Positive activity behaviours (i.e. higher physical activity [PA]/lower sedentary behaviour [SB]) are beneficial from infancy, yet evidence suggests that young children (0‐ to 6‐year‐olds) are relatively inactive. To better understand the perceived influences on these behaviours and to aid intervention development, this paper systematically synthesizes the extensive qualitative literature regarding perceived barriers and facilitators to PA and SB in young children (0–6 years old). A search of eight electronic databases (July 2016) identified 43 papers for inclusion. Data extraction and evidence synthesis were conducted using thematic content analysis, underpinned by the socio‐ecological model (i.e. individual, interpersonal, community, organizational and policy levels). Parents, childcare providers and children perceived seven broad themes to be important for PA and SB, including the child; the home; out‐of‐home childcare; parent–childcare provider interactions; environmental factors; safety; and weather. Each theme mapped onto between one and five levels of the socio‐ecological model; barriers and facilitators at the interpersonal level (e.g. parents, care providers and family) were most frequently cited, reflecting the important (perceived) role adults/peers play in shaping young children's behaviours. We provide an overarching framework to explain PA and SB in early childhood. We also highlight where gaps in the current literature exist (e.g. from male carers; in developing countries; and barriers and facilitators in the environmental and policy domains) and where future quantitative work may focus to provide novel insights about children's activity behaviours (e.g. safety and weather).

**Abbreviations**
ASSIA: Applied Social Sciences Index and Abstracts
BNI: British Nursing Index
CINAHL: Cumulative Index to Nursing and Allied Health Literature
MVPA: moderate‐to‐vigorous physical activity
PA: physical activity
PROSPERO: International Prospective Register for Systematic Reviews
SB: sedentary behaviour
SEM: socio‐ecological model
TV: television

## Background

Physical activity is beneficial to health and well‐being across the life course 1. Although the evidence base is well established in adults and school‐aged children 1, 2, physical activity also appears to be beneficial for very young children: in infants, toddlers and preschoolers, higher levels of physical activity are related to better social and motor development, improved metabolic health and decreased adiposity 3. In contrast, sedentary behaviour (defined as any waking activity characterized by an energy expenditure ≤1.5 metabolic equivalents *and* a sitting or reclining posture 4) is associated with higher adiposity and poorer psychosocial health and cognitive development in children 0–4 years old 5.

In recent years, interest in both physical activity and sedentary behaviour during the early years has increased. Prevalence estimates suggest that preschool‐aged children engage in low levels of physical activity and are sedentary for a large proportion of their day 6, 7. This sedentariness appears to manifest early in childhood, with children spending large amounts of time watching television (TV) before the age of 2 8. A wide range of interventions have therefore been developed in an attempt to boost activity levels and decrease sedentary time in young children prior to their entry into formal schooling 9, 10, 11. Such interventions often target a range of health behaviours (i.e. diet and physical in/activity) 10, 11, 12 or aim to increase physical activity/decrease sedentary time in isolation 9. Yet regardless of emphasis or setting, few studies report evidence of a positive effect on physical activity; those that do see small gains (<10%) in overall physical activity, which are not sustained over the longer term 9, 10, 11.

The reasons for lack of intervention success are likely to be varied, and in no small part due to the difficulties associated with changing behaviour 13. However, it has been suggested that for a greater chance of intervention success, it is important to establish the relevant influences on the target behaviour 14. To this end, several quantitative systematic reviews have been conducted to determine correlates (i.e. cross‐sectional factors) associated with young children's physical activity 15, 16, 17, 18 and sedentary behaviour 19. For both behaviours, these investigate a broad range of potential correlates including demographic, biological, environmental, social and psychological influences. Contrasting conclusions regarding what factors are related to physical activity behaviour tend to be drawn across reviews 15, 16, 18, but family factors 15, 16, 17, time spent outdoors, and the built or physical environment 15, 16 appear to be consistently associated with increased physical activity in preschool‐aged children. Evidence of the correlates of sedentary behaviour is less well elucidated, with many (early) studies tending to report TV viewing as a proxy for sedentary behaviour rather than using objective measures 19. In a review of correlates, child sex was assessed in four or more studies 19; it showed no association with TV viewing and an indeterminate association with accelerometer‐measured sedentary behaviour. Child's age, body mass index, parental education and race also showed an indeterminate association with TV viewing, whilst outdoor playtime was found to be not associated with TV viewing 19.

It is however difficult to draw firm conclusions about causality from cross‐sectional studies. As part of series of reviews, of which this qualitative synthesis is one, we assessed the quantitative determinants (i.e. longitudinal predictors) of physical activity in both prospective and intervention studies 11. Although 14 determinants were assessed in four or more studies, only parental monitoring and provider training were associated with positive change in children's total physical activity and moderate‐to‐vigorous physical activity (MVPA), respectively 11. A further 12 factors, some of which have also been identified as correlates of physical activity (e.g. sex and portable equipment), showed inconsistent or no association with change in preschoolers' physical activity over time. These quantitative reviews do however highlight that studies have tended to focus on the influence of specific domains (namely, child, family and environmental) on children's physical activity and sedentary behaviour. A large number of as‐yet unexplored factors (particularly in the community and policy domains) therefore remain, which when targeted may have the potential to effect positive change in young children's activity behaviour at a population level.

Drawing on qualitative research may be one way to explore these hitherto unmapped influences on young children's physical activity and sedentary behaviours. Much work has been conducted to explore what factors those caring for children (e.g. parents and caregivers), and the children themselves, perceive to be important for activity behaviour, but no systematic synthesis of this growing body of evidence has been conducted to date. Exploring and integrating this qualitative literature, particularly in combination with previous quantitative reviews, will enhance our understanding of the influences on young children's activity behaviours. Crucially, it may also lead to identification of potential avenues for intervention that those who are instrumental to children's activity behaviour believe to be important but that have yet to be explored by researchers and policy makers as important components of interventions.

This systematic review therefore aims to synthesize the qualitative literature exploring barriers and facilitators to activity behaviours (i.e. physical activity and sedentary behaviour) in young children (0–6 years), in order to (i) establish perceived influences on activity behaviours and (ii) consider where discrepancies and gaps in the wider (qualitative and quantitative) evidence exist that may inform future research.

## Methods

This review was conducted as part of a suite of reviews aiming to establish the determinants of obesogenic behaviours in children 0–6 years (including fruit and vegetable intake; sugar sweetened beverages and unhealthy diet intake; and sedentary behaviour). The protocol for this 20 and a quantitative physical activity review 11 have been described previously; the International Prospective Register for Systematic Reviews registration number is CRD42012002881. This review was carried out in three stages 21, 22, according to criteria for the rigorous conduct and reporting of systematic reviews 23. Studies were identified in tandem across all reviews, with smaller teams leading on data extraction for specific health behaviours of interest.

### Generic review methods

#### Study identification

We conducted a systematic search (common to all reviews) in August 2012, with four sets of search terms relating to the study population, study design (including qualitative studies), outcome of interest and exclusion of clinical populations (Table S1). An extensive scoping phase was conducted prior to the full search to maximize sensitivity and specificity. We searched eight electronic databases (MEDLINE, Embase [via OVID], CINAHL, PsycINFO [via Ebsco], Web of Knowledge [via Thomson Reuters], British Nursing Index, Applied Social Sciences Index and Abstracts and Sociological Abstracts [via Proquest]). We used endnote citation management software (Thomson Reuters, Philadelphia, PA, USA) to download relevant references, and the references of included papers and relevant reviews were subsequently searched. We did not limit included papers by language, but they were limited to published full texts. An updated search, identifying qualitative studies describing barriers and facilitators of physical activity and sedentary behaviour only (i.e. not including dietary behaviour search terms), was conducted in July 2016.

### Study selection

After an initial fidelity check between reviewers 11, three reviewers leading reviews on the included behaviours (K. H.: physical activity and sedentary behaviour) screened approximately 12,500 titles each. A small amount of basic information (e.g. study type and behaviour) about each study deemed to meet the inclusion criteria was extracted. Two random 5% samples of papers (total *n* = 3600) were also double screened by two additional reviewers (R. L. and E. V. S.) as a quality check. Relevant full texts were obtained and distributed for the behaviour‐specific reviews, with data extraction then occurring in parallel.

### Methods for activity behaviour qualitative review

#### Inclusion/exclusion criteria

Qualitative studies were eligible for inclusion if they provided an in‐depth discussion of barriers and/or facilitators to physical activity or sedentary behaviour in children between 0 and 6 years of age. Studies conducted with parents, caregivers (i.e. grandparents and childcare providers/educators) and children themselves were considered. Exclusion criteria included (i) studies using quantitative methods, including those conducting analysis of free text in questionnaires and (ii) studies conducted in clinical populations (e.g. children who were malnourished or had asthma, cerebral palsy, cystic fibrosis, autism, etc.). For mixed‐methods studies (e.g. qualitative and quantitative/q‐sort methods), the qualitative element of the study was considered if presented separately. As studies focused on the general population, parents and care providers of children with certain conditions (e.g. asthma) may have been included. Although the influence of clinical conditions on activity behaviours was not the focus of individual studies, if perceived to be important by parents and care providers, it would be drawn out in the extracted barriers and facilitators.

#### Quality assessment

A standard tool, specific to qualitative study designs, was used to assess the quality of included studies 24. Each was judged according to 12 pre‐defined quality criteria: (i) research question clearly stated; (ii) approach appropriate for the research question; (iii) qualitative approach clearly justified; (iv) context/(v) role of the researcher/(vi) sampling methods clearly described; (vii) sampling strategy appropriate for the research question; (viii) method of data collection clearly described/(ix) appropriate; (x) method of analysis clearly described/(xi) appropriate; and (xii) a sufficiency of evidence to support study conclusions (Table S2). Studies were scored out of 12, where high quality is ≥10; medium, 9–6; and low, ≤5. This was used as an indication of study quality but was not taken into consideration for the synthesis, as directly reported quotes/results were used for the thematic analysis here.

#### Data extraction and synthesis

All full texts identified for inclusion were read by K. H. and double screened for inclusion by E. V. S. Standardized forms were used to extract relevant information from each paper including the following: first author; publication year; country; study design, setting and population; and descriptive characteristics of the sample. Study‐specific data extraction and analysis were then conducted using a thematic framework approach, underpinned by the socio‐ecological model (i.e. individual, interpersonal, community, organizational and policy levels). Data extraction and synthesis proceeded in five stages. First, all papers citing potential barriers and facilitators to physical activity were read twice by K. H. Second, within each paper, key themes identified by original authors and/or this review team, and direct quotes relevant to these, were extracted. The extracted quotes were subsequently used to illustrate each theme; in some instances, quotes have been shortened here for brevity. Third, an iterative process was used to develop the emergent (or sub‐) themes across all papers. Fourth, these emergent sub‐themes were summarized and consolidated into summary themes, which were mapped onto levels of the socio‐ecological model. Finally, using the summary themes, K. H. and E. V. S. derived the overarching theoretical framework, again mapping this onto the socio‐ecological model. Any disagreement was resolved by consensus.

## Results

**Figure 1:**
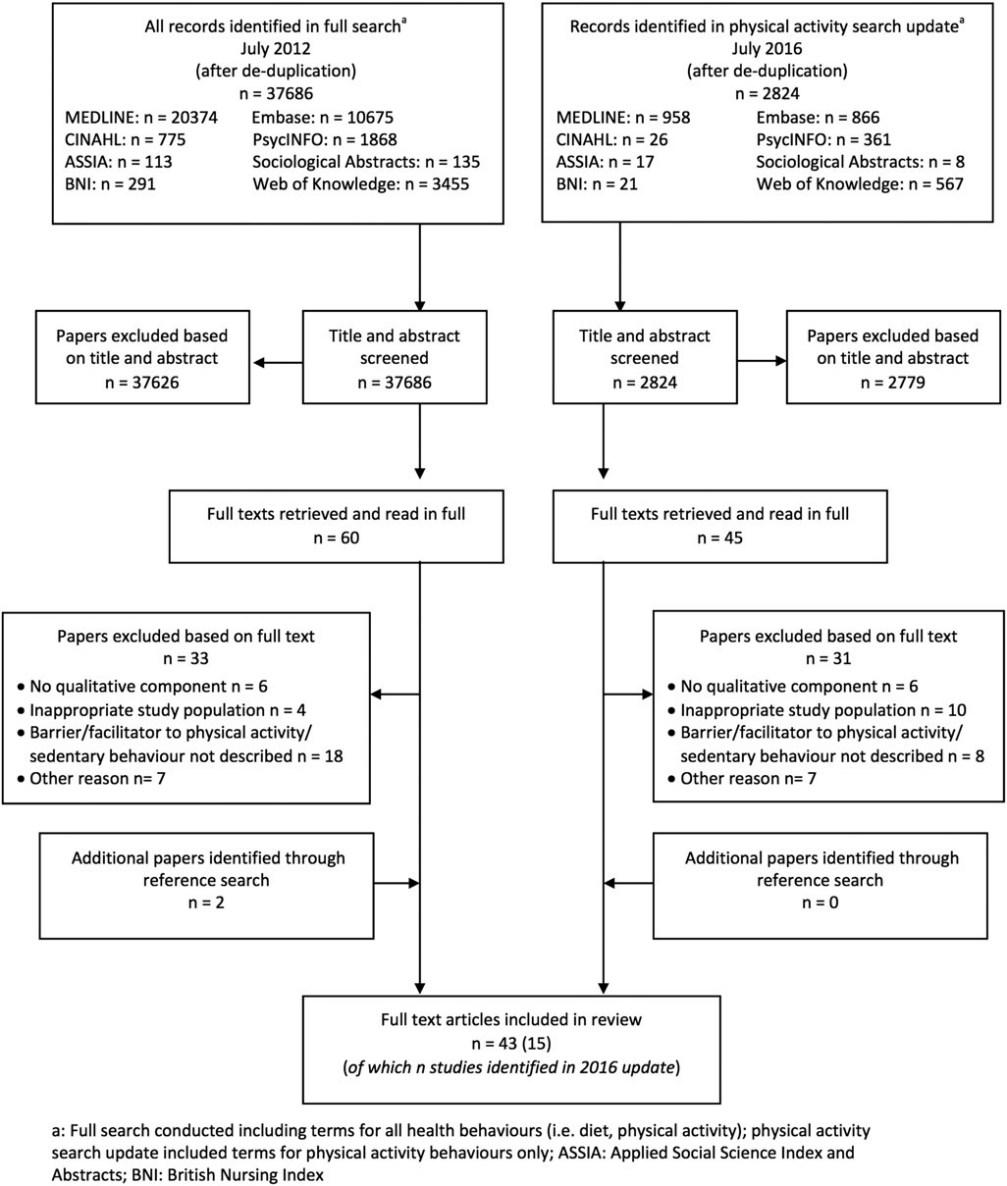
Flowchart outlining identification of papers for inclusion.

A total of 37,868 and 2,824 references were retrieved in 2012 and 2016, respectively, of which 220 were read in full and 43 qualitative papers (describing 35 study samples) relating to children's physical activity and sedentary behaviour were identified for inclusion (Fig. 1). A brief descriptive summary of studies is included in Table 1 (with a more detailed summary of each included study provided in Table 2). Studies were predominantly conducted in the USA, Australasia and Europe, and all bar one paper were published in or after 2004.

**Table 1:** Qualitative study characteristics

Sample characteristic	Study ID	Total number of studies (%)
Total sample size		
<50	25, 26, 27, 28, 29, 30, 31, 32, 33, 34, 35, 36, 37, 38, 39, 40, 41, 42, 43, 44, 45, 46, 47, 48, 49, 50, 51	27 (63)
50+	52, 53, 54, 55, 56, 57, 58, 59, 60, 61, 62, 63, 64, 65, 66, 67	16 (37)
Sample population		
Parents only	26, 28, 29, 30, 31, 32, 33, 34, 35, 37, 42, 49, 50, 51, 53, 56, 57, 58, 59, 60, 61, 62, 66	23 (53)
Care providers only	27, 36, 38, 39, 44, 47, 48, 54, 64, 65	10 (22)
Children only	45, 46, 55, 63, 67	5 (13)
Parents and care providers	25, 33, 41, 43, 52	5(13)
Method of data collection		
Focus groups	26, 27, 31, 35, 36, 40, 42, 43, 44, 46, 50, 52, 53, 54, 56, 57, 58, 59, 60, 64, 65	19 (44)
Interviews	28, 30, 32, 34, 44, 45, 47, 49, 62, 63, 66	11 (26)
Other	25, 29, 33, 38, 39, 41, 48, 55, 61, 63, 67	13 (30)
Country		
Australasia	29, 37, 43, 44, 53	5 (13)
Europe	25, 26, 28, 32, 46, 52, 54, 59, 62, 66	10 (22)
Africa	33	1 (2)
Canada	30, 31, 42, 57, 58, 61, 64, 65	8 (19)
USA	27, 34, 35, 36, 38, 39, 40, 41, 45, 47, 48, 49, 51, 55, 56, 60, 63, 67, 68	19 (44)
Quality		
High (≥10)	28, 30, 31, 32, 33, 34, 35, 36, 37, 38, 39, 40, 41, 42, 43, 44, 45, 46, 47, 48, 50, 53, 54, 57, 58, 60, 62, 64, 65, 66, 67	31 (72)
Medium (6–9)	25, 26, 27, 29, 49, 51, 52, 55, 56, 59, 61	11 (26)
Low (≤5)	63	1 (2)

**Table 2:** Summary of included studies

Author, publication year, region, country, study	Population	Sample characteristics	Recruitment	Data collection	Analysis	Author conclusions	Study quality
Rothlein 1987 FL, USA 63	103 preschoolers (parents, care providers excluded)	Mean age 2–6 years; ethnicity not stated; range of socioeconomic backgrounds	Drawn from private and public preschools and childcare centres of a variety of socioeconomic and cultural backgrounds in Dade County, FL	Children interviewed	Responses were tabulated and categorized by the researchers	Parents and teachers did not regard play as important for young children. Parents lacked interest in having their children play during preschool time, whilst care providers perceived adults were a major factor in limiting children's play	4
Goodway 2005 Midwest, USA 55	59 (30F) preschoolers	Mean age 4.74 years; African–American; mean income is $23,694, and 80% are on welfare	Children enrolled in KATCH, a compensatory preschool programme in 3 schools, with poor readiness for school	Used constant comparisons to compile various forms of data collected into a coherent structure	Inductive coding for topic themes and regularities. Data also triangulated using difference forms of data	No real conclusions drawn	9
Irwin 2005 Ontario, Canada (sample as in Tucker 2006) 57	71 (68F) parents	Age range 21–63 years, with approximately 60% in their 30s; 95.5% Caucasian, 1.5% South East Asian, African–American, other; 42% worked full time, 23% part‐time and 35% were unemployed	Recruited through flyers, information sheets and site visits at community locations (5 playgroups, 3 day care centres, 1 resource centre and 1 workplace). 2 of the 10 sites were located in rural areas	10 semi‐structured FGs, 4–11 people in each	Inductive content analysis to code and categorize emerging themes	Need for education and interventions that address current barriers are essential for establishing PA as a lifestyle behaviour during early childhood and, consequently, helping to prevent both childhood and adulthood obesity	10
Tucker 2006 Ontario, Canada (sample as in Irwin 2005) 58	71 (68F) parents	Age range 21–63 years, with approximately 60% in their 30s; 95.5% Caucasian, 1.5% South East Asian, African–American, other; 42% worked full time, 23% part‐time and 35% were unemployed	Recruited through flyers, information sheets and site visits at community locations (5 playgroups, 3 day care centres, 1 resource centre and 1 workplace). 2 of the 10 sites were located in rural areas	10 semi‐structured FGs, 4–11 people in each	Inductive content analysis to code and categorize emerging themes	Increased parental awareness of current programmes/resources currently available; modification of schedules to include morning and afternoon sessions; physicians to hand out PA ideas/resources, particularly during the winter	10
Pagnini 2007 New South Wales, Australia ‘Weight of Opinion Study’ 37	32 (32F) parents	Age range 20–49 years; from predominately English‐speaking backgrounds; 62% employed at least part‐time; educational attainment varied from school university qualifications	The directors of the centre/preschools distributed a recruitment flyer, information sheet and consent form to the parents of all of the enrolled children. Parents registered with the researchers to participate in the group	7 FGs: 3 with parents from log day care, and 4 from parents of preschools	Thematic analysis to identify overarching themes between groups	Mothers' perceptions about their children's eating and weight are emotionally intense: interventions need to go beyond information and engage with parents' emotions. Prevention efforts need to acknowledge the issues faced and provide support: making healthy and active behaviours easily available/providing local services	10
Pagnini 2007 New South Wales, Australia ‘Weight of Opinion Study’ 44	11 care providers (6 from long day care, 5 from preschools)	No information about age, ethnicity and SES provided	10 managers approached to participate	Individual interviews bar one	Thematic analysis to identify overarching themes between groups	Dissemination of educational resources/professional development opportunities to support staff to promote activity. Developing national guidelines would also be helpful	10
Bolling 2009 Midwest, USA 50	22 (19F) parents; 1 grandmother	Parents were 37.1 years (28.6–45.1 years, with children 3.6–6 years); non‐Hispanic, Caucasian; all were generally college educated and would be considered middle class (Hollingshead class III 1⁄4 3, class IV 1⁄4 13, and class V 1⁄4 7); 2 were parents on Medicaid	Via letter and then telephone from a paediatric practice serving sub/urban and rural patients in Suburban Midwest (10% Medicaid, 10% AA and 5% Hispanic)	FGs	Consensus ratings among 3 coders to understand the depth and breadth of information discussed using 4 classes: question/prompt, major themes, minor themes or other topic	Parents want paediatricians to speak clearly about weight status, explain rationale for concern, relate that concern to family history and provide specific advice/treatment recommendations	12
Ceglowski 2009 MN, USA 45	29 preschoolers (10 < 2.5 years; nineteen 2.5–4 years)	Mean age 2–6 years; 13% spoke home language other than English; SES not stated	Purposeful selection according to representativeness of population included in study, range of ages and number of children in the family, type of childcare used and eligibility for childcare assistance. Staff sent letter translated into 6 languages to all families receiving Child Care Assistance. Interested families completed a card and mailed it back to the researchers	Home interviews	Themes were extracted and pictures were also examined to incorporate into the major themes	Contributes additional insights to the limited literature on children's perceptions of childcare	10
Copeland 2009 OH, USA (sample as Copeland 2011, 2012) 48	48 (48F) care providers	Age not stated; providers had worked in childcare settings between <1 and 37 years; 28 (55%) identified themselves as African–American; 44 (90%) had at least some education beyond high school	Maximum variation sampling using flyers and several community agencies to recruit a heterogeneous convenience sample of childcare providers	9 FGs, and then thirteen 1‐2‐1 interviews	Inductive approach to identify emergent themes	Inappropriate clothing (of a few children) may preclude PA (for the majority) in childcare. Clear and specific policies for required clothing required so that children's active play opportunities are not curtailed. Parents may need education about the importance and benefits of active play for children's development	12
Ferrarri 2009 Ontario, Canada 42	Parents	Mean age not stated; Tamil/Chinese; most had held professional jobs previously, 2 had same job in Canada, remaining mother were underemployed or unemployed	Immigrant parents from 2 distinct ethno‐communities (Tamil and Mainland Chinese) within the Greater Toronto Area. The sampling strategy was purposive rather than random (mixed purposeful sampling vs. randomized sampling), to receive the maximum range of information possible	6 FGs with mothers – 3 with each group of Tamil and Chinese mothers	Inductive data analysis to delineate themes and interpret meaning of the data extracted	Parents from different ethno‐cultural backgrounds should be directly involved in the development, implementation and evaluation of interventions in the future	10
Hessler 2009 CO, USA 41	12 (12F) parents; 13 informants	No information about parental age, ethnicity and SES provided	Resident in the rural county (parent of PSC) and able to read, write and speak English	Parents and informants participated in interviews or FGs	Identification of key themes and patterns acknowledged by coding for organization, retrieval and interpretation of data	Rural areas may not be as conducive to everyday PA for children as traditionally believed	10
Nuananong 2009 rural, agricultural state, USA 40	10 (8F) parents	Median age of parents 30 (range 25–35); ethnicity not stated; 70% worked full time, 20% part‐time and 10% unemployed	Recruited through newspaper and public postings. Eligible participants were parents living with overweight preschool child 3–5 years (BMI > 85th percentile). The parents spoke English and provided written consent for participation in the study	1 FG with mothers and fathers	Thematic content analysis	Results have implications for health professionals in planning/developing educational materials for PA interventions, enhancing motivation for activity of rural populations and working towards the reduction of barriers through policy and relevant resource acquisition. Continued research required to provider culturally appropriate ways to increase PA within a rural population	12
Pagnini 2009 New South Wales, Australia ‘Weight of Opinion Study’ 43	32 (32F) parents; 11 (11F) care providers	Age range of mothers: 20–49 years; mix of cultural backgrounds; 62% employed full time/part‐time and most from rural/low–medium SES metropolitan areas	4 areas in Sydney and outside were chosen and schools/GPs approached. Parents were subsequently approached through schools	Parents: 7 FGs; care providers: 11 interviews	Thematic analysis to identify overarching themes between groups	Obesity is a complex public health issue, and commonality of views is important. However, this could be used to collaborate across groups	10
van Zandvoort 2010 Ontario, Canada 64	54 (54F) care providers	29% of care providers were <25 years, 16% >45 years; 85% Caucasian; 96% had college or greater education	Via 17 organizations providing day care for toddlers and preschoolers in the London (Ontario) area	8 FGs with 6–8 pts	Inductive content analysis to identify themes	Provides contextual and descriptive information with implications for directors, parents and researchers to promote and support PA participation among preschoolers in day care	11
Akhtar‐Danesh 2011 Ontario, Canada 61	33 parents	Mean age of parents was 34.4 years; ethnicity not stated; most educated to college/university level	Phase 1: convenience sample of parents attending a medical centre in Canada for their well‐baby check‐up to establish/compile Q‐sample statements to cover major views of parents and to be used for phase 2. Phase 2: A convenience sample from the same clinic	Parents were classified into 2 groups according to confidence in delivering ‘healthy nutrition’ ‘family physical activity’	Q‐sort	One‐third believed PA benefitted children and did not see being overweight or obese as a barrier to PA. Parents were well educated but further education required about providing integrated nutritional and PA in school curriculum and increasing PA time	9
Cammisa 2011 Italy ‘PERISCOPE’ 46	49 preschoolers	Children's average age was 4 in class A and 5 in classes B and C; Italian; SES not stated	Recruited through 3 different kindergartens reporting to the same Central School Institution	FGs took place in the classroom the children usually play in, in one big group	Summarized answers, classified them into movement/sedentary activities as well as hindering/promoting factors reported	The use of drawings reliable and easy tool to understand children's PA habits. There is a need to change the beliefs and the behaviours of care providers and parents who seem to be non‐architectural ‘invisible’ barriers. Children want more resources at KG, such as portable and table games	11
Copeland 2011 OH, USA (sample as Copeland 2009; 2012) 69	49 (48F) care providers	Age not stated – participants had worked in childcare settings (range < 1 to 37 years). 28 (55%) identified themselves as African–American, 44 (90%) had at least some education beyond high school	Maximum variation sampling using flyers and several community agencies to recruit a heterogeneous convenience sample of childcare teachers, securing a small sample of great diversity	9 FGs, and then 1–2–1 interviews	Inductive approach to identify emergent themes	Children could have very different gross motor experiences even within the same facility (with presumably the same environment and policies). This is based on the beliefs, creativity and level of engagement of their care provider (who has many different roles, which impact children's activity)	12
Lanigan 2011 WA, USA 67	81 preschoolers	Mean age of children 3–5 years; 44% of eligible children from minority cultures; 58% of population were girls	Sampling frame consisted of 663 children aged 3–5 who attended for‐profit and government‐supported or community‐supported not‐for‐profit childcare centres, college lab schools and family childcare homes	Child role play addressing dietary feeding practices and barriers to PA; proceeded to interview. Each session took 20–30 min on average	*A priori* theory guided the development of the initial coding scheme used to analyse the child role play/interview, with continuous revision as new themes emerged. When a pattern emerged, the new theme was integrated into the coding system	Children demonstrated better understanding of the benefits of healthy eating compared with PA. Obesity prevention efforts targeting young children need to use consistent messaging across all contexts. Key gaps in young children's understanding include: the importance of drinking water, that snacks are part of nutritional intake and the benefits of engaging in PAs	10
Sansolios 2011 Denmark ‘PERISCOPE’ 25	Preschoolers; parents; care providers	Mean of children was 4–5 years, ethnicity and SES not stated	KGs recruited from the 14 Danish KGS part of the PERISCOPE project. Method piloted in 1 KG	Interviews, drawings, observation; FGs for adults	—	The new methodology of videotaping gives the researcher the chance to interpret a wider range of responses. However, this method contains a weakness, if used alone as it only reflects what the video camera has recorded	7
Stenhammar 2011 Sweden 26	30 (25F) parents	Of the sample, 20% were 20–30 years, 50% 31–40 years and 30% 41–50 years; 73.3% born in Sweden; 10% completed compulsory schooling, 53% high school and 37% university/college	Participants were selected from respondents to a previous questionnaire study regarding family stress and children's BMI; random sample of 80 parents was selected from the original sample. Inclusion criteria: child born in 2004 and had participated in the earlier questionnaire study. Only one parent per child invited those with disabled children excluded. 8 non‐native Swedish parents were invited through a preschool (but had participated in earlier survey)	5 FG: 4 with randomly invited parents (2 with higher‐educated and 2 with lower‐educated parents); 1 FG with purposeful sample	Systematic text condensation. Context of each theme sorted into categories describing aspects of a theme	Parents struggled to give their children a healthy lifestyle, with ‘temptations’ of daily unhealthy choices causing hassles and conflicts. Parents desired professional support from preschool, Child Health Care and a collective responsibility from society with uniform guidelines. Parents groups were mentioned as peer support	9
Tucker 2011 Ontario, Canada 65	84 (83F) care providers	Mean age 33 years; 87% Caucasian; the majority of participants (79%) had a college education, and an additional 13% had either a university or post‐graduate degree	Staff were drawn from 3 organizations that ranged in the number of facilities within the city (i.e. 1, 12 and 13 centres across London)	8 FGs with providers from 1 organization; additional 5 FGs provided in‐depth understanding of the importance childcare providers–parent interactions	Inductive content analysis to identify themes	Highlights the need for increased parent–caregiver partnering in terms of communication and cooperation in service of promoting appropriate amounts of PA among preschoolers	11
Copeland 2012 OH, USA 39 (sample as Copeland 2009, 2011)	49 (48F) care providers	Age not stated, but participants had worked in childcare settings between <1 and 37 years; 28 (55%) identified themselves as African–American; 44 (90%) had at least some education beyond high school	Maximum variation sampling using flyers and several community agencies to recruit a heterogeneous convenience sample of childcare teachers, securing a small sample of great diversity	9 FGs, and then 1–2–1 interviews	Inductive approach to identify emergent themes	Societal priorities for young children – safety and school readiness – may hinder children's physical development. When designing environments to promote optimal health and development, holistic thinking required about potential unintended consequences of policies	11
De Decker 2012 Belgium, Bulgaria, Germany, Greece, Poland and Spain ‘TOYBOX’ 59	122 parents	Age range 23–50 years; multi‐ethnic across countries; low and medium SES	Parents of medium–high SES were recruited through preschools, kindergartens or through researchers' networks. Specific recruitment strategies through community centres for low SES citizens or through charity institutions (e.g. in Belgium)	24 FGs (2–10 people in each; 2 low and 2 medium SES in each country)	Content analysis	Parents should be provided with alternatives for screen activities and information on how to set rules for screen time to assist in decreasing children's screen time	9
Hesketh 2012 Victoria, Australia ‘InFANT’ 53	95 Parents (I: 61 [61F] and PSC: 34 [32F])	Mothers of infants had median age: 32 years (21–38) and mothers of preschoolers: 38 years (28–71); born in Australia: I: 54 (89%), PSC: 27 (75%); degree: I: 40 (66%), PSC: 12 (33%)	First‐time parents of infants (<12 months old) and parents of preschool‐aged (3–5 years) children from 1 socioeconomicallyand ethnically diverse local governmentarea in met Melbourne, Australia. First‐time parents were from maternal and child health centres (*n* = 8) and preschool parents recruited from randomly selected preschools	FGs: no formal questions were used – participants brainstormed topics that came to mind	Grounded theory; using thematic analyses to extract themes conveying main messages	PA is a mixture of innate and family driven; there are many barriers and facilitators for PA, which differ by child's age. New parents were optimistic regarding their ability to positively influence their child's PA and screen time; such optimism was not apparent among parents of preschool children	10
Bellows 2013 CO, USA 51 ‘Food Friends’	24 (24F) parents	Mean age 35–44 years; 96% Caucasian; 75% had college degrees	Purposive sampling from existing parenting groups from the Colorado area	FGs based on a structured script of open‐ended, probing questions	Common ideas and themes were identified, based on the number of responses per category, as well as descriptive quotations	Mothers were more receptive to the term ‘gross motor development’ rather than PA. Parents may feel responsible for the gross motor development of their child, yet they perceive PA as ‘natural’. Physical activity messages need to be targeted to resonate with parents	7
De Craemer 2013 Belgium, Bulgaria, Germany, Greece, Poland and Spain ‘TOYBOX’ 52	122 parents; 87 care providers	Mean age of parents 32–38.4 years; mean age of care providers 34.2–46.5 years; ethnicity not stated; 36% low SES parents	Recruitment in municipalities with the highest prevalence of overweight or obesity of either child or parent (ToyBox intervention taking place in comparable municipalities)	4 parent and 3 care provider FGs conducted in each country	Content analysis; including quotes and excerpts from all 6 countries using qualitative data analysis	Parents and carers believed children to be sufficiently active, and that it was important for children to learn to sit still etc. Many barriers and facilitators to PA suggested	7
De Decker 2013 Belgium, Bulgaria, Germany, Greece, Poland and Spain ‘TOYBOX’ 54	87 care providers	Mean age of 22–59 years across all countries; multi‐ethnic across countries; conducted in low/medium SES areas	Teachers in the 6 European countries were recruitedthrough preschools or through researchers' networks	18 FGs	Content analysis	Interventions should focus on increasing care provider awareness of how sedentary preschoolers are during the preschool day. Care providers should be informed about strategies to sedentary time	10
O'Connor 2013 TX, USA ‘Ninos Activos’ 56	74 (68F) parents	Mean age not stated; Hispanic – 82% participated in Spanish‐speaking group; family income of under $20,000/year (55%); 45% reported having at least a high school diploma	Convenience sample of parents recruited via fliers posted or distributed at local community centres, churches, health fairs, food fairs and retail outlets in Houston, TX; notices posted on the Baylor College of Medicine and USDA/ARS Children's Nutrition Research Center volunteer websites; calls to research volunteers listed on the CNRC research volunteer database	5 FG talking about promoting PA (3 groups reported higher education); 5 talking about barriers to PA (2 groups reported higher education).	NGT – a structured multi‐step group procedure that can elicit and prioritize responses from a group of people in reaction to a question or problem	Parents identified ways to encourage and discourage 3‐ to 5‐year‐olds from PA – both are important targets for interventions. Further research required to determine the role parents play in discouraging children's activity, especially in using psychological control or submitting children to abuse	8
Carson 2014 Alberta, Canada 31	Parents	Mean age of parents was 36 ± 6 years (of children of mean age 33.5 ± SD 14 months); approximately 30% of those children had a younger sibling and approximately 33% had an older sibling; ethnicity not stated; the majority of parents were mothers (85%) who were married (89%) and had a graduate degree (74%)	Parents were primarily recruited through information letters, newsletters and presentations at parent meetings	7 semi‐structured FGs	Thematic analysis (but not specifically called this)	Gain‐framed messages around the role of screen‐based and non‐screen‐based SB for children's cognitive and social development might be most effective for adoption of the guidelines. Providing parents the guidelines early with resources for minimizing SB should be considered. Research is needed in other demographic groups of parents to confirm these findings	10
Lyn 2014 GA, USA 47	20 care providers (directors)	Mean age and ethnicity not stated; 37% had earned a high school diploma or GED, 26% held a graduate degree, 21% held a bachelor's degree and 16% held an associate's degree	To participate in the programme, centres were required to be licensed by the state and not be located in an elementary school. The programme included 58% (*n* = 14) for‐profit and 42% (*n* = 10) nonprofit centres	20 semi‐structured interviews	Not explicit – thematic/coded analysis	Nutrition and PA policy changes perceived to be beneficial to the childcare environment. Highlights important considerations for efforts to promote healthy weight environments in the early care setting	11
Bentley 2015 South West England, UK (sample as Bentley 2016) 32	24 parents	Mean age of parents not stated (but their children were 2 [11.1%], 3 [63%] and 4 [25.9%]); ethnicity not stated; recruitment from 1 urban neighbourhood from each of the first, second and third tertiles of the IMD within the City of Bristol, UK and one rural neighbourhood 13 km south of Bristol (second tertile of IMD) were targeted	Study information given via posters and leaflets to preschools, day nurseries and mother and toddler groups located within these areas at least 1 week prior to recruitment. Mothers approached face‐to‐face either during the group time or at child pick‐up/drop‐off time	24 semi‐structured interviews	Thematic analysis; framework analysis	Mothers do not identify with the need to increase PA or reduce SB in their child – awareness of activity guidelines alone is unlikely to initiate behaviour change. Information about accurately assessing PA and SB should be provided with guidelines. Clear messages need to be developed that reframe the guidelines into pragmatic and usable targets	11
Birken 2015 Ontario, Canada ‘TARGet Kids’ 30	14 (11F) parents	Mean age of parents not stated (but their children were on average 31 [17] months); they had a total of 12 siblings with a mean (SD) age of 67 (49) months; ethnicity not stated; 13 (93%) children had mothers with college/university‐level education	Via two sites of TARGet Kids!, a primary‐care, practice‐based research network in Toronto. English‐speaking parents of ambulatory children aged 1–5 years already recruited to TARGet Kids! were approached at their child's well‐child visit	14 semi‐structured interviews	Thematic analysis	Parents do not consider the sedentary nature of strollers. Researchers interested in PA promotion in the early years might consider strollers and the context of their use in developing and testing strategies to promote PA and reduce SBs	12
Buro 2015 Midwest, USA ‘Active Where?’ 49	15 parents	Mean age of parents not stated (but their children were 3.6 ± 0.74 years); 100% Caucasian; 40% rural	Recruited through a network sampling method using word of mouth or a flier sent home with children enrolled in Head Start sites located in rural eastern North Dakota	15 semi‐structured interviews	Constant comparison, or simultaneous data collection and analysis	Public transportation solutions and enhanced neighbourhood safety are potential community‐wide obesity prevention strategies in rural communities. Interventions should be tailored to community stage of readiness. Strong social networks should be considered an asset for community change in these regions	8
Edwards 2015 South West England, UK ‘B‐Proact1v’ 66	53 parents	Mean age was 37.5 ± 5.92 years; 86% of the sample was Caucasian British; 23% were unemployed or full‐time parents with 77% in full‐time or part‐time work	Parents whose children were participating in existing study: children who provided ≥3 valid days ofaccelerometer wear time and an address and postcode (to allow for calculation of SES) were included in the sampling frame for interviewing	53 telephone interviews	Deductive content analysis	Friends and siblings influence young children's PA and screen viewing behaviours. Child‐focused PA and screen viewing interventions should consider the important influence that siblings and friends have over these behaviours	12
Lindsay 2015 MA, USA 36	44 (41F) family care providers	Mean age not stated; Hispanic/Latino; *n* = 14 had graduated from high school or earned their GED, *n* = 17 had attended some college	Random selection of 22 names on list of FCCPs per region of the state. All selected providers were emailed a recruitment flyer in Spanish that included a phone number that interested providers could call to obtain more information and/or express interest in participation	6 FGs	Content analysis	Latino family care providers can have a strong impact in promoting healthful behaviours in low‐income, Latino communities. Potential to effectively deliver interventions targeting low‐income, minority families to promote healthful eating and PA behaviours and prevent child obesity	11
Martin‐Biggers 2015 NJ and AZ, USA 60	139 parents	Mean age of parents was 32.18 ± 7.12 years, and they had 2.29 ± 1.15 children; 40% Spanish speakers; about two‐thirds had received at least some college education (*n* = 47 high school or less; *n* = 48 some post‐secondary; *n* = 42 baccalaureate or higher; *n* = 2 no response)	Recruited via flyers posted at community sites and emails sent from workplace Listservs in New Jersey and Arizona	10 FGs addressing PA	Content analysis	Future education programmes with preschool parents should emphasize support and encourage sharing of helpful strategies among parents	11
Pulakka 2015 Mangochi district, Malawi ‘iLiNS‐DOSE’ 33	Parents; nurses	Mean age of parents was 29 years; Malawian; 2 illiterate	Convenience sampling to identify parents of young children from different socioeconomic backgrounds and age groups. 2 key informants interviewed who had deep knowledge on the culture and child health in the study area through their long careers as a nurse	15 in‐depth interviews; 1 FG; 2 key informants	Inductive content analysis	Malawian parents' concept of children's PA is more comprehensive than scientific definition and includes aspects of both physical and mental activity	12
Suen 2015 Hong Kong 29	45 parents	Parental age not stated (but their children had median age 4 years); 47/57 spoken Cantonese; 28 participants had a median monthly household income >$HK20,000	Purposive sample of kindergartens, preschool playgroup centres and maternal and child health centres of the Department of Health stratified by area socioeconomic status (low to middle and middle to high) of their location	NGT: 6 FGs and 12 individual interviews	Inductive thematic analysis	Parental practices that encourage or discourage preschoolers' PA were identified. These can assist with development of a culturally sensitive parenting practices scale and inform future quantitative research	9
Tovar 2015 RI, USA 27	30 family care providers	Mean age was 50 years; Hispanic (predominantly Dominican: 77%) and Spanish speaking; 50% had at least some college education or a college degree or higher	R2LP recruited providers for the formative research. To be eligible to participate in the FGs, providers needed to be current FCCPs for children ages 2–5, speak English or Spanish, and be at least 18 years old	4 FGs	? Thematic analysis	Family care providers are aware of the importance of healthy eating and PA: need to address the specific barriers they face/operationalize their knowledge into practical everyday actions. Data will inform development of a culturally relevant, multicomponent intervention for ethnically diverse family care providers	9
Woo Baidal 2015 MA, USA 35	49 parents	Mean age of 17 pregnant parents: 25.6 ± 6.4; 15 with infants: 25.6 ± 7.5; 17 with children in early childhood: 27.9 ± 6.1; Hispanic parents, with Spanish only: 9, 4 and 3 from each group; high school grad: 13, 9 and 12 in each group	Women with a prenatal visit at federally qualified community health centre in Boston and singleton gestation eligible for pregnancy groups; parents (mothers or fathers) of children between birth and 6.9 months eligible for infancy groups; those with children of age 7–24 months eligible for early childhood groups	7 FGs (2 pregnancy, 3 infants, 2 early childhood)	—	Opportunities exist in the first 1,000 days to improve Hispanic mothers' understanding of the role of early‐life weight gain and other risk factors for obesity. Interventions that link health care and public health systems and include extended family may prevent obesity among Hispanic children	12
Zahra 2015 South West England, UK ‘B‐Proact1v’ 62	50 parents	Average age of mothers was 38.8 ± 5.7 years, 11% had 1 child, 62% 2 children and the remaining 27% had 2 or more; predominantly Caucasian British (89%); 19% of participants were unemployed/full‐time parents, 62% worked part‐time and 19% worked full time	Participants whose child provided at least 3 valid days of accelerometer wear time, a valid postcode and address to allow for calculation of SES and consent to be contacted were included in the sampling frame for interviewing	50 telephone interviews	Deductive content analysis	Fathers play a key role in children's PA choices and behaviours; they can influence children in a variety of ways. Parents tend to share in the PA‐related tasks of their children, but father availability seems to be a factor in their amount of involvement. Improving child PA may benefit from developing interventions that target both children and fathers	10
Bentley 2016 South West England, UK (sample as Bentley 2015) 28	26 parents	Age of parents not given (but their children were 2 [13.8%], 3 [51.7%] and 4 [34.5%] years); ethnicity not stated; recruitment from 1 urban neighbourhood from each of the first, second, and third tertiles of the IMD within the City of Bristol, UK and 1 rural neighbourhood 13 km south of Bristol (second tertile of IMD) were targeted	Information about the study was given via posters and leaflets to preschools, day nurseries and mother and toddler groups located within these areas at least 1 week prior to recruitment. Mothers approached face to face either during the group time or at child pick‐up/drop‐off time	26 semi‐structured interviews	Thematic analysis; framework analysis	Mobile device use by preschool children is common. More research is needed to determine the impact of this; how much time preschool children spend using mobile devices; and which activities their use may be replacing	11
Grzywacz 2016 NC, USA 34	33 parents	Age not stated; Latino; SES information not provided	Purposive sample of mothers of children in farmworker households balanced by farmworker status (i.e. seasonal vs. migrant), child age (2–3 and 4–5 years of age) and child gender. Those in network of community contacts serving either seasonal or migrant farmworker families referred potential participants to study staff	33 semi‐structured interviews (17 migrant, 16 seasonal)	Not explicit – thematic/content analysis	Both the built and social environments pose several barriers to children's PA, exacerbated by cultural beliefs. Active play/PA promotes good health and equips children to work hard, but too much active play or intense PA is potentially dangerous. Children also perceived to be sufficiently active, and sedentary forms of play benefit children's brains	10

AZ, Arizona; BMI, body mass index; CNRC, Children's Nutrition Research Centre; CO, Colorado; F, female; FCCP, family childcare provider; FG, focus group; FL, Florida; GA, Georgia; GED, general equivalency diploma; GP, General Practitioners; IMD, Index of Multiple Deprivation; KATCH, Keeping all the children healthy; KGs, Kindergartens; KGS, change to KGs; MA, Massachusetts; MN, Minnesota; NC, North Carolina; NGT, nominal group technique; NJ, New Jersey; OH, Ohio; PA, physical activity; PSC, Preschool children; RI, Rhode Island; SB, sedentary behaviour; SES, socioeconomic status; TX, Texas; USA, United States of America; USDA/ ARS, U.S. Department of Agriculture Agricultural research service; WA, Washington State.

Across papers, 77 barriers and facilitators to physical activity and sedentary behaviour were reported (Table 4). Most studies used focus groups and semi‐structured one‐to‐one interviews conducted with childcare providers and parents of preschool‐aged children (the majority of whom were female) to elicit responses. A few studies also explored the views of preschool‐aged children through drawing and discussion. Thematic/content and inductive analysis were most commonly used to analyse the data. In general, studies tended to include predominantly lower socioeconomic or racial minority groups. Many of the included papers (*n* = 31 [70%]) were deemed to be of high quality: most adequately explained the rationale for their approach, justified the methods used and explained the type of analysis conducted, although studies were often likely to draw conclusions that were not always fully supported by sufficient evidence.

### Barriers and facilitators to physical activity

**Figure 2:**
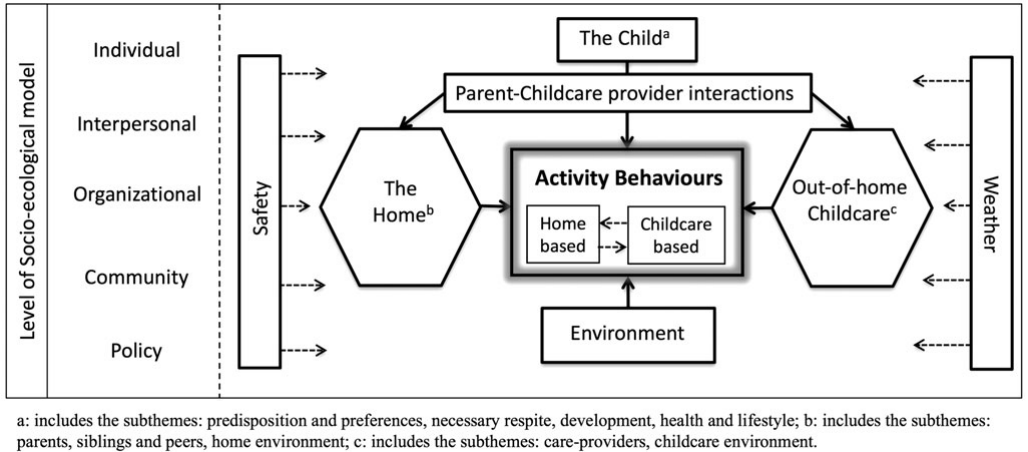
Overarching theoretical framework.

The derived theoretical framework is shown in Fig. 2. Barriers and facilitators to children's physical activity were classified into seven broad thematic areas: the child, the home, out‐of‐home childcare, parent–childcare provider interactions, environmental factors, safety and weather. These themes spanned one (i.e. child within the individual level), several (i.e. the home/out‐of‐home childcare across interpersonal, community and organizational levels) or all (i.e. safety) levels of the socio‐ecological model. A synthesis of the findings within each thematic area is provided later (also see Table 3 and 4).

**Table 3:** Summary of factors influencing young children's activity behaviours

Broad themes (and sub‐themes)	Studies exploring each (sub) theme
The child	
Pre‐disposal and preferences	25, 27, 28, 29, 30, 31, 32, 33, 42, 52, 53, 54, 56, 57, 58, 59, 60, 65
Necessary respite	27, 28, 29, 31, 32, 33, 34, 35, 36, 45, 54, 56, 59, 60
Development	26, 30, 32, 34, 37, 39, 52, 53, 54, 55, 56, 57, 63
Health and lifestyle	33, 53, 54, 56, 61
The home	
The role of parents	26, 27, 29, 30, 31, 32, 33, 34, 37, 40, 41, 42, 43, 44, 49, 52, 53, 55, 56, 57, 58, 59, 60, 62, 65
Siblings and peers	29, 33, 34, 41, 42, 45, 49, 52, 53, 56, 57, 59, 62, 65, 66
The home environment	34, 36, 52, 53, 55, 56, 58, 59, 60, 65
Out‐of‐home childcare	
The role of care providers	25, 27, 36, 38, 44, 46, 52, 54, 64
The childcare environment	27, 36, 39, 40, 44, 47, 48, 52, 54, 55, 56, 61, 64
Parent–provider interactions	26, 27, 29, 33, 36, 37, 41, 43, 46, 47, 48, 49, 52, 53, 56, 62, 65
The environment	26, 27, 31, 32, 34, 37, 41, 44, 49, 52, 54, 57, 58, 60, 62
Safety	27, 29, 37, 38, 39, 41, 44, 46, 49, 52, 53, 54, 56, 58, 61, 64
Weather	27, 31, 34, 36, 38, 40, 49, 52, 54, 57, 59, 60, 64, 67

**Table 4:** Barriers and facilitators to 0‐ to 6‐year‐old children's activity behaviours

Determinant	Influence	Reported by		
		Children	Parents	Carers
The child				
Pre‐disposal and preference				
Sex (female)	−		25, 32, 33, 52, 53	54
Age/differing age groups	−		57	27, 54
Ethnicity/culture	−		42	
Innate ability	*±*		32, 52, 53	
Child preferences	*±*		56	27, 54
TV/electronic media	*±*		28, 29, 30, 31, 32, 33, 52, 56, 58, 59, 60	65
Necessary respite				
Downtime required			28, 29, 31, 32	54
Not all SB time equal			29, 31, 32, 34, 35, 59, 60	27, 36, 54
Restraint not seen as SB			32	
No further capacity			32	
Perception that PA will cause trouble			29, 33, 34, 60	27
Naughty behaviour	−	45	56	54
Development				
Early habit formation	+		37, 53, 57	
Skill level	−		53	54
Child enjoyment of PA	±	55	26, 30, 32, 57	52
Structured activity			37, 52, 53, 56, 57	
Designated play times	+		56	
Independent exploration	*+*		52, 53	
Letting them be children/age appropriate activities	±		56	
Academic prioritization	−	63	34	39, 52
Health and lifestyle				
General health	±		33, 53, 56	
Obesity	−		56, 61	54
Lifestyle (diet, sleep)	±		33, 56	
Regular sport and exercise	+		61	
The home				
The influence of parents				
Active parents/role modelling	±	55	26, 31, 33, 37, 40, 41, 42, 52, 53, 57, 59, 62	65
Parental employment	−		29, 34, 53, 56, 60, 62	
Parent–child participation	+		29, 32, 52, 53, 56, 60, 62	
Parent lack of energy/ill health	−		29, 32, 40, 52, 53, 60	
Parent–childcare collaboration	+		52	52, 65
Cost of activities	−		29, 37, 40, 52, 56, 57, 60	44
Parental supervision			29, 34, 49	
Appropriate scheduling	±		40, 53, 58	
Needing to do chores			29, 59, 60	27
Encouragement/active alternatives			29, 62	
Lack of time	−		26, 29, 31, 32, 34, 40, 43, 52, 53, 56, 57, 60	
Increased parental awareness	+			65
Instilling values about PA	±		53, 56	
Information for HCPS			31, 49, 60	
Use of motorized transport	−		52, 56	
Use of stroller (when too big, for safety)	−		30, 31, 32, 33, 56	
Logistic support			29, 62	
Siblings and peers				
Siblings			52, 57, 59, 66	65
Playing with friends	+	46	33, 34, 41, 42, 52, 56, 66	
Coming together as a family/on holiday	+		29, 53, 56, 62	
Social support – friends with children	+		49, 52	
Home environment				
Internal home environment	±	55	52, 53, 56, 58	65
Outside space/at home	±		34, 52, 53, 56, 59, 60	36
Active transport/walk to school	+		53, 58	52
Attending a school nearby	+		53	
The childcare centre				
Childcare provider	±	46		25, 27, 36, 38, 44, 52, 54, 64
Provider allergies	−			38
Low teacher self‐efficacy	−			38
Childcare environment				
Time spent in childcare	±		40	39
Active opportunities at school	+		56, 61	
Space at preschool	±	55		36, 39, 52, 54, 64
Resources (in preschool)	±			27, 44, 47, 48, 52, 64
Music	+			64
Group activities	+			52
Parent and care provider interaction				
Childcare as an initiator of PA	+	46	26, 33, 37, 41, 43, 49	65
Parent support/encouragement/PA instigation			26, 29, 52, 53, 56, 62	65
Interactions between parents/providers				27, 36, 47
Clothing	−			48
Community				
Resources (in community)	±		34, 37, 41, 52, 60	44
Built environment			31, 49	54
Rural location			34, 41, 49, 52	44
Community support	+		41	
Changing society	−		26, 41, 57	
Social environment/stranger danger			49, 60	
Location of PA programmes	−		58	
Improved (access to) facilities	+		58	
Time outdoors			32, 49	27
Organized activities			32, 49, 60, 62	27
Safety				
Perceived safety risk	−	46	29, 37, 41, 49, 53, 56, 58, 61	27, 39, 44, 54, 64
Perceived health risk (parent/carer)	−	46		38
Safety/policy Legislation	−			39, 52, 64
Weather	±	67	31, 34, 40, 49, 57, 59, 60	27, 36, 38, 52, 54, 64
Air pollution			34	

HCPS, healthcare professionals; PA, physical activity; SB, sedentary behaviour.

### The child

#### Pre‐disposal and preferences

At the individual level, parents described activity as being innate, with girls perceived to be naturally less active than boys 25, 52, 53. Enjoyment of activity was also perceived to facilitate physical activity by care providers 52, 54, and children themselves 55.

I think they have got this natural desire to run around and play, I don't think you have to do too much to encourage them. (Parent [P]) 53

It's absolutely important. And with my 7‐year‐old it's not a problem. It's hard to keep him indoors, but my 5‐year‐old, she's more of an indoor gal. (P) 68

A child's own preferences for more sedentary behaviours were cited as largely negative influences by parents 26, 56, 57 and care providers 27, 54. Parents and carers also stated that children generally had a preference for use of electronic media over more active pursuits 28, 29, 30, 31, 52, 56, 58, 59, 60. Other adults did however state that children could be active or indeed be encouraged to be more active when in front of the TV 29, 32, 33.

Children can spend all day watching television nowadays. My daughter is completely spellbound in front of the television. It is not possible to talk to her. This is often a struggle, sometimes I just switch off the TV. Unfortunately, not always …. (P) 26

#### Necessary respite/downtime

This theme encompasses parents' 28, 29, 31, 32 (and care providers' 54) widely expressed belief that children require a certain amount of daily downtime. Importantly, not all sedentary time was perceived to be equal, with reading and time spent sitting devoted to academic and development activities deemed to be essential 27, 29, 31, 32, 34, 35, 36, 54, 59, 60, even if this was in front of a screen.

Sometimes to calm her down we sit at the table and I'll teach her something … putting puzzles together, stuff like that … well is sitting and colouring really a sedentary behavior? Or playing with Play‐Doh? … I think those creative activities are important. (P) 31

I rather think that I have to slow down my child at that age. … He should not get too much [activity] … he is only 4. (P) 52

#### Development

Parents mentioned the importance of positive early habit formation 37, 53, 57, and a higher skill level (i.e. both motor skills and skills relating to physical activity more generally) in facilitating higher activity levels 53, 54.

If you start them young, then they'll build a lifestyle to carry them right through their entire life. (P) 57

We have to go out of our way to teach them sports because we have found if you don't … you get to a certain age where they just won't do it. … and they won't play because they are the only ones who don't know how to … (P) 53

Parents also thought that regular 61, structured activity 37, 52, 53, 56, 57 was (on the whole) related to higher activity levels in their children. Age‐appropriate activity and allowing children to grow up and undertake independent exploration were further ways that carers suggested activity may be facilitated 52, 53, 56.

You know like if they've got swimming lessons, I'm happy to take them, but [father name] is like really kind of on it that they need to get the lessons. (P) 62

[Children's physical activity] is a necessary element as far as what we were saying earlier, their muscle development and their balance and I think there is a lot of social development that goes along with it too. (Childcare provider [CP]) 38

However, there was also recognition of the need for children to be ‘school‐ready’, with academic development often deemed to be (more) important for young children 39, 52, 63.

Because even though I feel that the gross motor is something that's important for the children to experience and engage in, I don't think that their parents necessarily do … I don't think that physical activity is high on the priority list of things that schools want to necessarily provide. (CP) 39

I wish he'd sit down and write his name and all that because that would make him learn more things, because doing physical activity makes them develop physically, but they should also develop their ability to pay attention. (P) 34

#### Health and lifestyle

Children with a healthier lifestyle 33, 56 (i.e. good diet and much sleep) were perceived to be more active, with a child's general health cited as positive and negative influences, where children with better health 53, 56 were more active. Overweight/obesity was universally described as a barrier to physical activity 54, 56, 61, where children with higher body mass indexes were thought to be less active or more sedentary.

I noticed that my child sleeps deeply when he plays hard like running all day. (P) 40

I think one important barrier to physical activity in obese children is that it is more difficult to participate in activities because of the obesity itself … I think that exercising and sports are very important to a child's health status. (P) 61

### The home

#### The role of parents

By far, the most commonly cited influences, both positive and negative, were at the interpersonal level. Both parents and childcare providers frequently stated that active parents, who could act as role models for children, facilitated young children's activity 26, 31, 33, 37, 40, 41, 42, 52, 53, 54, 57, 62. Parents participating in activities with their young child were also deemed to facilitate activity 29, 32, 52, 53, 56, 60, 62, with other positive parental influences including positive encouragement and support, and parents instilling the value of physical activity in their children 53, 56.

Parents are responsible for young children's lifestyle; they can't make their own choices, we have to make their choices …. (P) 26

I feel that adults play a crucial role in the physical activity of children. If adults after dinner sit in front of the TV, kids would sit in front of the TV as well. If you go out for exercises, they would follow you. (P) 42

If you're going to the convenience store, walk to the store … instead of taking the elevator, taking the stairs. So with my kids I try to do that in the mall, go up the stairs even though it takes a lot longer and walking to the store instead of taking the car. (P) 58

They did however acknowledge that they themselves may also be a barrier to children's activity 25, 38, 40, 42, 52, 53, 57, 64. For example, parents stated that their job 29, 34, 60, 62 resulted in a lack of time 26, 29, 31, 32, 34, 40, 43, 52, 53, 56, 57, 60 and energy 29, 32, 40, 52, 53, 60. They also had to make the effort to juggle multiple schedules (within the family) 40, 53, 58, balancing this with the cost of providing active opportunities 37, 40, 44, 52, 56, 57, which at times limited their young children's physical activity. These barriers also meant that parents tended to use less active modes of transport (i.e. stroller 30, 31, 32, 33, 56 and car 52, 56) as they were more convenient and often quicker, which further impacted their child's opportunities to be active.

My mom never does anything with me, she just takes me places and leaves me there. (Child (C)) 55

… I've just started working full‐time and it's a long commute and I'm away from the home for such a long period of the day that [I] do more with the kids on the weekend. Yeah, okay then between the laundry and the cleaning and everything else, especially at this time of year [December] when there's so much extra stuff to do that I can't even begin to think of doing some organized physical activity with the Kids. (P) 57

I think it gets to a point of how much can you actually do? You know I've got three kids, how many places am I supposed to be after school on one night? (P) 53

usually just to get anywhere, popping out [for] groceries … heading to the park … basically anytime we need to get somewhere with a fairly tight timeline [we use the stroller]. (P) 30

Related to this, a physically active child was often also deemed to have the potential to cause trouble 27, 29, 33, 34, 60: limiting physical activity was reported as one way to punish naughty behaviour 54, 56, with children themselves mentioning that bad behaviour meant they were prevented from being active 45.

Yeah, they [the children who are too active] are not controlled by the parents. So, it's not … it becomes a level whereby you start also [to] complain: no, this is now too much. (P) 33

For example, when asked how (bad) behaviour influenced physical activity:

One 4‐year‐old stated that you ‘get dead, time out’. A 3‐year‐old explained that another child ‘pushed me down. I cried. They got time out’. (C) 45

#### Siblings and peers

Children's wider social networks were also noted to be important: siblings were perceived to both facilitate and inhibit children's physical activity levels 52, 57, 65, with younger children often wanting to mimic or play with their older siblings. In general, interacting with friends was cited by children 46, and parents as facilitating physical activity 33, 34, 41, 42, 52, 56, 66. Parents believed that coming together as a family, either at home or on holiday, also positively influenced their young children's activity 29, 53, 56, 62.

They [siblings] go out in the garden and she will go and play with them, I suppose so. She doesn't tend to want to play on her own. In fact, I had to always pull her back because she looks up to her brother who is a very active boy and he has been going to all these things, and she always wanted to be just like him. (P) 66

If you live in a neighborhood with adults only, they would not have play‐mates and they would have less exercise. That is my case. If you live in a neighborhood with children who like to play with you, that is different. (P) 42

I have a lot of fun with my friends and I feel better. (C) 46

Well actually all of us together, me my husband and [Participating child] … my husband we always liked kickboxing, never had the chance to do it, so we thought oh we'll ask would you like to go and try. (P) 62

The influence of friends and peers was however limited to developing social interactions with children of the same age, with mixed age groups of children thought to hamper younger children's activity levels 27, 54, 57.

I want to do more group sports but I've got two different ages [of children] to worry about … Just more sports available for the younger 2 to 3‐year olds. (P) 58

#### The home environment

More broadly, living close to a child's childcare setting and therefore using active transport to get there (e.g. walking and cycling) was a perceived positive influence 53, 56, 61. Parents stated that a sedentary home environment, including a TV being persistently on 52, 56, 58, and small outside spaces inhibited activity 34, 52, 53, 59, 60. This said, both childcare providers and parents suggested that homes with adequate space and resources (i.e. active toys, etc.) facilitated activity in small children 52, 53, 56, 58, 64.

One of the priorities for me in terms of where we live will be related to school. I really will like to be close to a school so that we don't have to rely on cars. And we always walk to the local shops and to the hairdressers and things. (P) 53

We have a few parents that walk to school and home and I think that obviously encourages physical activity. (CP) 65

My son walks and jumps inside the house. But if you have a small house, the space, the room for him to exercise is limited. What I am saying is that it depends on the condition of your family. (P) 42

[We] live [in an] upstairs [apartment], so it's hard to have indoor playtime that won't bother the neighbors downstairs … it is very easy for things to get broken inside the house. (P) 60

### Out‐of‐home childcare

#### The role of care providers

Childcare providers felt that they were able to facilitate and encourage young children to be active 25, 27, 38, 44, 52, 64, which was corroborated by children themselves 46. Childcare providers 25, 38, 52, 64 did however acknowledge that they also may hinder children's activity, with barriers to providing active opportunities including the demands of work, the need to prioritize academic outcomes, wanting to prevent noise and their own reluctance to go outside 38, 39.

You know, It's all the teacher's decision of how much time they're gonna get and how much they're gonna do … Some teachers just aren't into that and some teachers are into that. So it depends. (CP) 38

Sometimes the children are easier to motivate when I participate myself. When I run around and jump on one leg they have a lot more fun and rather take part than when I just stand there and play with my drum. (CP) 52

Our teacher says that, if we run too much, we sweat, and thus we get a cold and we can no longer attend the kindergarten … our teacher says that we can not play outside football or basket, because we are too lively and we are at risk of hurting any other. (C) 46

I was born in the Dominican Republic. I am not used to the kind of cold weather we get here in [Massachusetts]. I do not think I will ever get used to it … I just try to get through the winter. It can be difficult. (CP) 27

#### The childcare environment

Space and resources within the childcare environment were often discussed as helping and hindering children's physical activity. This may be particularly important given childcare was perceived by providers to be the only place children could be active 38, 44. In general, larger spaces, curriculum materials and play equipment 27, 47, 52 were perceived to benefit children's activity levels. A lack thereof (i.e. small spaces 36, 39, 54, 55, 64 and poor resources 39, 44, 64) within a preschool environment was perceived to largely inhibit children's active opportunities.

So we provide opportunities for the children to get a bit active … I think it is important that we do that especially as the children who are here five days a week. If they don't get stuff like that at the weekend, at least when they come here, they are on the go all the time. (CP) 44

We've got more stuff [indoor play equipment] now, so whether it's too hot or too cold or too rainy outside, we take it out, then they can do it [activities] in the classroom. So, yeah, being that we have that stuff now, it does make it easier and they get to do a lot more [physical activity]. (CP) 47

My preschooler actually is in a [private preschool] program and they don't have the room to have the physical activity part of it every day or even once a week, so they get it once a month. (P) 57

You have 42 kids in the playground and you only have 10 bicycles so it's difficult … [s]o it [stems] from the [lack of] budget … because you go to these new centers and they have all [these] fancy‐dancy toys and we have some broken‐pedaled bikes. (CP) 64

### Parent and childcare provider interactions

Of particular interest was the persistent push–pull between parents and childcare providers regarding children's physical activity. As mentioned previously, care providers believed parents to be important role models for their children's physical activity, and responsible for inhibiting their child's active opportunities by, e.g. supplying inappropriate clothing 48 or being worried about children playing outside in inappropriate (i.e. usually cold and wet) conditions 36, 39, 46, 48. Conversely, parents saw childcare centres as a crucial initiator of physical activity for their in children 26, 33, 41, 43, 49, 52; the active opportunities provided by childcare centres were mentioned as being central to children's daily needs 56, 61.

There is no real point in teaching them here and they go home and they do something different so it is a two‐way thing! (CP) 43

I've had problems with parents telling the kids, ‘Don't get dirty!’ because of what they have on. So that's I guess a pet peeve of mine … They're going outside. They are going to play. They are going to be on the floor. They are going to be, you know, children. (CP) 48

I have a hard time when parents don't respect the rules that I have around children bringing and using electronics such as DS. I don't really like to have to keep reminding them that those devices are not allowed … Communication with parents is … very important because it gives us a chance to learn about the child's home environment, the family's routines and rules. (CP) 36

Nevertheless there was also acknowledgement of this tension, and both parents 52, 65 and childcare providers 27, 36, 47 stated that collaboration with the other party was vital for preschool‐aged children's active opportunities. Working together, with improved communication between parents and care providers, was often cited (by childcare providers) as how this might be achieved 27, 36, 47, 52.

There is no real point in teaching them here and they go home and they do something different so it is a two‐way thing! (CP) 43

[j]ust like what you do with us as educators, like professional development for us … it's kind of like for the families; if you could help to promote it or inform them [parents] then maybe they might go [to neighbourhood activities and programs] …. [i]f we don't educate the parents, how are we going to educate the kids. We need to work together with them. It's very important …. (CP) 65

Information that will help would be like to have more communication [with parents about the importance of physical activity] now that winter's coming. (CP) 27

Communication with parents is also very important because it gives us a chance to learn about the child's home environment, the family's routines and rules, which is really important information to have to understand and care for the child in our FCCHs. (CP) 36

### Environment

Resources within the community, including parks and playgrounds, presented both barriers 34, 37, 41, 60 and opportunities for activity 44, 52. Children spending increased time spent outside 27, 32, 49 and having community support for activity 41 available were perceived to be universally positive influences.

Because there is a lot of fear around with drug users and a lot of crime and that, people don't let their kids out. … I don't let my son out in this area by himself … so he is stuck inside a flat with me, and unless I take him outside, he doesn't get to go outside. (P) 37

Our town is a pretty secluded area and that helps because parents feel comfortable sending their kids outside, and you know they can do whatever they [parents] want in the house and they know that their kid will be ok. … The environment, it is a great place; there is so much to do outside. (P) 41

In order to prevent obesity in childhood, it is necessary to build inspiring playgrounds and green areas where children can play …. (P) 26

Interestingly, although the majority of parents living in rural locations thought this benefited their children's physical activity 41, 49, 52, rural locations may also prove to be barrier owing to their isolation and a lack of resources 34.

We used to go to the mall in [urban town] a lot because they had this little brand‐new indoor play area. There's nothing really like that indoors around here. Like, a play place for kids, you know? I feel like there are a lot of open places in [rural town] that you could fit something like that in. (P) 49

I haven't seen a park around here. Well, there is one in Wilmington, but it's too far to take them in the afternoon. It takes about an hour to go and come back. (P) 34

### Safety

A common theme mentioned by parents, care providers and even the children themselves was safety. This influence, which was predominantly mentioned as a barrier, spans all levels of the socio‐ecological model. Many parents worried that a physically active child could and would hurt themselves and may therefore be likely to limit their child's activity 29, 37, 41, 49, 53, 56, 58, 61. Children also mentioned that adults' fears in relation to their safety and their health limited their activity levels 46.

Mom says that I can play with the ball with my sister, but not with all my friends at the kindergarten, because she is afraid I can get hurt … once a friend of mine pushed me and I was hurt and my mom told my teacher that I had not to play running. (C) 46

I think that the most important barrier to physical activity in children is safety concerns re: letting kids outside un‐supervised. (P) 61

The way I see it, I prefer that they watch television rather than being endangered outside. (P) 56

This preoccupation with safety was also widespread in childcare providers 27, 39, 44, 54, 64, particularly in relation to restrictive preschool policies preventing children from being active 39, 52, 64.

I don't think they really get their heart rate up much from climbing because with all the new licensing regulations [in childcare centres], our climbing equipment isn't that hard anymore …. (CP) 39

We can go on walks except it's got to be a field trip so it's a little more difficult in the sense that we can't go for a neighborhood walk. It has to have a specific purpose, and then we have to get permission. (CP) 64

In order to offer moving opportunities, more staff is necessary. For instance, a climbing landscape requires more supervisory staff. The children are not allowed to do it by themselves. (CP) 52

Importantly, perceptions about changing societal norms and children no longer being allowed to or wanting to go out to play were cited by parents to be major barriers to their children's activity 26, 34, 41, 49, 57, 60.

I grew up in a country town where you could just go off and play. There were gangs of kids running around the street playing cricket and stuff. You're not living in the city and your kids are just free. (P) 53

This appeared to relate to both their local environment and parents' and children's circumstances: often parents expressed a fear that something might happen to their child if they did not watch them, they were concerned about danger in their surrounding area and, in some instances, that others would perceive them to be bad parents.

Because, sometimes, they fall or they can run away and get run over by a car. So, it's better inside because I can keep an eye on him, here in the room or in the hall: If they go out, they have to go out with us …. They told us that if they saw them playing by themselves there, they were going to be taken by social services. (P) 34

### Weather

Finally, and often mentioned in combination with safety, the weather was cited as both a facilitator (parents 34, 49, 57, 60 and childcare providers 38, 52) and more often as a barrier by children 67, by parents 31, 40, 49, 57, 59 and childcare providers 27, 36, 38, 54, 64 to young children's physical activity. At the extreme, this posed a perceived health risk for children, particularly when playing out in the cold 38, with ‘extreme heat’ and ‘mosquitos’ 60 also influencing physical activity at the other end of the weather spectrum.

During the summer their activity behaviors are great. I love how active they are and how they want to be outside doing things. (P) 57

Sometimes, I see on the news that children in the sun can pass out. I worry when the sun is very strong because if it's very hot, her heart can beat faster and she could pass out or something. (P) 34

I think the cool air. In the wintertime, (imitates a parent saying) ‘Oh no, don't go outside because it's cold outside’. I think a lot of people have the misconception that you are going to get sick if you go outside in cold weather. Really, it's better for you. (CP) 38

No, my mom does not want me to play outside at the kindergarten, because otherwise I get sick. (C) 46

Children urged the dolls to stay inside saying, ‘No, that would make them wet’. (C) 67

When it is raining outside or it is very cold, then the children are inside the school building because we [the preschool] do not have a covered playground. And then we [teachers] put on a movie. (CP) 54

## Discussion

To our knowledge, this is the first review to synthesize the qualitative literature relating to barriers and facilitators to activity behaviours in children 0–6 years old. Barriers and facilitators were classified into seven broad themes: the child, the home, out‐of‐home childcare, parent–childcare provider interactions, environment, safety and weather. These themes spanned between one and all levels of the socio‐ecological model (Fig. 2); although themes were categorized according to a primary focus, these themes are not mutually exclusive within the socio‐ecological model (SEM), highlighting the complex interplay between barriers and facilitators of young children's activity behaviours. Parents, care providers and the children themselves most commonly cited influences at the interpersonal and organizational levels as barriers and facilitators. A large number of factors remain unexplored in the qualitative literature in the community and policy domains. Evidence from this synthesis does however provide new as‐yet unexplored avenues for intervention (e.g. parental time and resources, available space, weather and safety). Moreover, targeting factors that those caring for young children, and the children themselves, believe to be important may enhance intervention tailoring, ultimately effecting both greater increases in young children's physical activity and decreases in sedentary behaviours.

This review combines qualitative evidence relating to barriers and facilitators to both physical activity and sedentary behaviour (together termed ‘activity behaviours’). Whilst researchers often differentiate between these behaviours for research purposes, many of the participants in studies included here failed to make any distinction: e.g. a barrier to physical activity was often perceived to facilitate sedentary behaviour (e.g. TV viewing). This was true of both parents and care providers, suggesting that those looking after young children perceive these behaviours to be equal and opposite. Moreover, guidelines for young children recommend specific amounts of physical activity, whilst limiting sedentary time (e.g. 2). Therefore activity behaviour can be thought of as occurring on a spectrum, where sedentary behaviour and very vigorous physical activity lie at opposite ends. This evidence synthesis is therefore beneficial to researchers working in the fields of both sedentary and physical activity behaviour, providing novel information about what those who are most influential to young children's activity behaviours perceive to be important.

### Barriers and facilitators to young children's activity behaviours

As noted earlier, a wide range of facilitators and barriers to activity behaviour were identified in the qualitative literature that have yet to be explored quantitatively 11. Interestingly, parents (and care providers) frequently expressed the belief that children require a certain amount of daily downtime and should not be constantly active. Moreover, not all sedentary time was perceived to be equal, with more academic or developmental activities (e.g. reading and crafts) deemed to be essential in contrast to TV viewing. This fits with the oft‐mentioned perception that children are naturally active, which may result in parents seeking to balance this with more sedentary (calming) activities. In addition, many parents mentioned that allowing their children to engage in more sedentary behaviours for part of the day has the added benefit of allowing them to go about their chores during an already hectic day; parents may therefore also benefit from their child's downtime, providing them with some necessary respite to ‘get things done’.

Physical activity guidelines for toddlers and preschool‐aged children (i.e. ambulatory infants to age 5 years) in several western countries state that children should accrue 180 min of any intensity activity above sedentary 2, 70, 71. Studies suggest that children may meet these guidelines 72, 73, in part owing to a large proportion of time spent in light intensity activity 73. Allowing a certain amount of ‘down time’, which in many cases was stated to be of educational value in the qualitative literature here, may therefore be reasonable. Nevertheless, it is important to distinguish between developmentally beneficial (i.e. reading, drawing and crafts) and non‐beneficial or entertainment‐based sedentary behaviours (e.g. TV viewing), as recently acknowledged by the American Association of Pediatrics. Their guidelines for screen time published in 2016 state that for children age 2 to 5 years, ‘entertainment‐based’ screen time should be limited to 1 h per day, and that infants aged 18 months or younger should not be exposed at all 74; screen time for educational purposes is not included in this time, but some media use for social interaction (e.g. Skype and FaceTime) is permitted. Clearly, younger children will likely benefit from screen time promoting pro‐social behaviour. However, parents frequently acknowledged that media use was a major barrier to their children's physical activity 28, 29, 30, 31, 52, 56, 58, 59, 60. Providing strategies to reduce entertainment‐based screen time in favour of other developmentally appropriate activities may help parents reduce their reliance on sedentary behaviours involving entertainment‐based media.

More generally within the home theme, many parent‐level barriers, including parents lacking time 26, 40, 43, 52, 53, 56, 57 and resources 52, 53, 56, 58, 64, were perceived barriers to physical activity in preschool‐aged children. Although it is not possible to, e.g. increase space within the home or provide parents with more hours in the day, it may be feasible to provide parents with ideas about how to, e.g. use their available time or space more creatively. Use of wider environmental resources, such as parks and community space, which were deemed to positively influence children's physical activity behaviours, may also help parents and children to actively interact. This may be particularly beneficial to boost activity within families 75. In addition, practical advice would be helpful given the concerns parents expressed about child safety when being active and young children's reliance on parents to take them to these places.

The qualitative literature here suggests that within the childcare centre, care providers perceive themselves to be important for children's physical activity. Quantitative literature suggests that providing training for childcare providers may influence change in children's MVPA, but the precise mechanism for this is not clear given the wide variation in training across interventions and countries 11. As no quantitative studies to date have specifically focused on care provider (physical activity) behaviour as a potential determinant of children's activity behaviour 11, it is difficult to determine the direct role childcare providers play in influencing preschoolers' physical activity. Moreover, qualitatively, providers did acknowledge that their own behaviour may on occasion inhibit preschool‐aged children's physical activity 38, 52, 64. This was evidenced in one cross‐sectional study conducted in Belgium preschool‐age children, where fewer supervising teachers during recess was associated with higher step counts in girls, but not boys 76. Consequently, childcare providers' own behaviour, e.g. preferring to stand or sit during children's outdoor playtime 76, may inadvertently reduce children's and girls' activity in particular. Future work should therefore focus on providers' own behaviour in relation to the children in their care. In addition, given the widespread preoccupation identified with young children being school‐ready, work exploring how physical activity may be integrated into academic tasks across the day, as a learning tool rather than separate need, may prove beneficial to children's activity. Such work would be timely in light of provider perceptions about their ability to encourage children to be active, and given the current growing interest in this sphere of research for children later in childhood 77.

Several cross‐sectional quantitative reviews conducted previously suggest that elements in the preschool environment may be positively associated with children's activity 78, 79. Childcare providers frequently stated that available resources and space within the childcare environment were both positive and negative influences on preschoolers' activity in the qualitative literature 39, 44, 52, 64. However as yet, no clear association between the childcare environment and change in physical activity has been found in interventions 11. In order to show exactly what influences children's physical activity in this setting, it may therefore be beneficial to assess the way children *interact* with the staff themselves and the environment, rather than focusing on the role of specific elements within the environment.

### Emerging themes for use in intervention development

The perceived influence of parent–childcare provider interactions on children's activity behaviours was a novel and potentially important finding to emerge here. Whilst parents clearly have a significant role in shaping young children's health behaviours, young children now spend increasing amounts of time in out‐of‐home care 80. It is perhaps unsurprising then that parents, care providers and the childcare environment were deemed to be important initiators of young children's physical activity, or that interventions to increase physical activity have also often simultaneously targeted both home‐based and childcare‐based elements. However, few to date have incorporated or assess interactions *between* these elements. Moreover, given the opinions expressed here, that each of the other party (i.e. parents and care providers) may be thought to be responsible for children's physical activity, exploring the exact nature of possible conflicts between parents and care providers could provide vital information about how best to encourage physical activity in home and childcare settings. Interventions focusing on interactions between parents and childcare providers (and their environments) could therefore feasibly have a significant positive influence on children's activity behaviours, with resolution of between‐group tensions further augmenting intervention success.

Across the quantitative literature, factors such child and parental knowledge have frequently been targeted as potential determinants of physical activity but are rarely associated with positive change in young children 11. Interestingly, these often‐targeted determinants were not mentioned across qualitative studies as barriers and facilitators to physical activity, suggesting that adults (and also children) are already have implicit knowledge that physical activity is beneficial. In contrast, the two final themes identified in this review, safety and weather, were largely cited as barriers to children's physical activity. Yet despite both being plausible and potentially influential determinants of young children's activity behaviours, they have rarely been explored in the quantitative literature 11, 16, 18.

Extreme weather was frequently mentioned in the context of child safety, but a general preoccupation with ensuring children were protected, usually at the expense of being physically active, was pervasive. In childcare centres, this risk aversion was commonly linked to policies around play and requirements for establishing ‘safe’ environments before children could be allowed to be active 39, 52, 64. Indeed, children themselves perceived their play was restricted because physical activity could result in them hurting themselves 45. Notably, the terms ‘play’ and ‘activity’ were often used interchangeably across studies here. Play is described as ‘some social, locomotor, fantasy, or object‐directed activity that is not directly functional’ 81, which a child usually engages in voluntarily 81. It can occur in each of four domains (i.e. locomotor, object, pretend and social) and can be, with the exception of social play, enacted alone or in a group 81. Children accumulate their physical activity in each of these domains, and largely in an informal manner during the preschool years 82. Changing societal norms noted here, tending towards risk‐averse behaviours that limit children's activity and opportunities to take exploratory risks, may consequently impact not only children's physical literacy/activity 83 but also their social and motor development through play 82. Modification of adult risk perceptions may therefore be one way to positively influence young children's activity behaviours, risk awareness and wider development. Doing so, either by intervention or in natural experiments, e.g. changing health and safety practices, would be an interesting and hitherto unexplored avenue of research.

Moderate weather, either hot or cold, was perceived to facilitate children's activity behaviours 34, 38, 49, 52, 57, 60; hot and cold extremes were perceived here to result in less physical activity and more sedentary time in preschoolers. This fits with quantitative research assessing weather as a correlate of physical activity. For example, in locations with more temperate weather year round 84, 85, samples of US preschoolers tended not to show variation in physical activity. In contrast, in studies conducted in regions where clear seasonal variation in weather exists, there are comparable fluctuations in physical activity (e.g. in samples of UK 73, 86 and New Zealand 87 preschool‐aged children). This was also shown in a longitudinal study of 3‐year‐old children conducted in Cincinnati, USA, where hot and cold weather conditions were negatively associated with total activity and MVPA, and positively associated with sedentary time 88. Where possible, learning to adapt to weather conditions that may provide fun opportunities for physical activity (i.e. wet weather), or providing parents and care providers with viable contingency options in locations were weather extremes are common, may be advantageous. This could prevent such marked seasonal variation in young children's physical activity and lead to consistently higher levels of physical activity in this age group year round.

### Social context and future research directions

The studies included in this review were from culturally diverse sub‐populations, and frequently recruited from lower socioeconomic strata. In general these groups are less likely to participate in quantitative (both prospective and intervention) studies. It is therefore beneficial to ascertain the perceived barriers and facilitators to activity behaviours across strata qualitatively. This work suggests that the concerns and activity promotion ideas of those caring from preschool‐aged children (i.e. safety, the influence of the home and childcare centres) are relatively stable across infancy and the preschool‐period despite differing socio‐demographic profiles. Moreover, parenting during the early years is time intensive regardless of resources. Therefore, that consensus as to the barriers and facilitators to young children's activity behaviours emerged across several themes here hints to a number of determinants of children's activity behaviour being viable future intervention targets. Importantly, based on this work, such suggestions as to how to promote activity behaviours could potentially be externally valid across preschool‐aged populations internationally.

Finally, this review highlights were gaps in the current (qualitative) literature exist. Interestingly as in the quantitative literature 11, there was a paucity of research with fathers and male care providers, and in developing countries; greater engagement with these populations is required as perceptions and influences on young children's physical activity may differ by carer sex and cultural practices. In addition, all included studies used focus groups or (semi‐)structured interviews to elicit participant responses. Other qualitative data techniques (such as ethnography/observation) may also provide valuable insights into how parents/carers and young children operationalize perceptions around activity behaviours in daily life, creating an important bridge between qualitative and quantitative literature. Further, in an era after the epidemiological transition 89, it is more challenging to determine why some children are more or less active than others as we often lack the heterogeneity in exposures (and outcomes) to explore associations quantitatively. Developing countries are currently undergoing similar transitions towards lower physical activity and higher sedentary time as those seen previously in higher income countries, perhaps at a fast rate. By conducting both qualitative and quantitative research in developing countries, where greater heterogeneity may still exist, we can ascertain potential influences on young children's activity behaviours. From this, we can learn how best to reverse negative trends towards inactivity in the future. Lastly factors in the environmental (community resources, rural locations and changing society) and policy domains of the SEM were rarely studied in the quantitative literature, but were suggested by parents and care providers in the qualitative literature as having a significant impact on young children's activity behaviour. These factors therefore represent key future avenues for quantitative activity promotion research in the preschool years.

### Strengths and limitations

This review is the first to specifically explore and systematically synthesize the qualitative barriers and facilitators to activity behaviours in children aged 6 and under. We applied rigorous review methods and did not exclude papers based on time of publication or language (although no foreign language papers were identified). In addition to a systematic literature search, we used a parallel reference examination to yield an additional two studies for inclusion. As this review was restricted to published studies, publication bias cannot be discounted. However, that we included a range of international studies, which used differing methods of qualitative data collection and analysis, is another strength of this review.

Qualitative studies were predominantly published from 2005 onwards, with a large increase in studies after 2012. These studies, conducted across differing continents, provide only a snapshot in time of parental, childcare provider and, to a lesser extent, children's activity behaviour perceptions. A large number of qualitative studies (72%) were classified as high quality, adequately explaining the rationale for their approach, justifying the methods used and explaining the type of analysis conducted. This said, included studies tended to draw conclusions that were not always supported by sufficient evidence (i.e. did not explicitly state methods such as triangulation were used to confirm findings), or made generalizations to populations not included in the study sample.

Qualitative research enables in‐depth insight into specified topics of interest, with authors interpreting evidence and subsequently using methods such as triangulation and member checking to confirm the credibility (or ‘truth’) of their findings 90. This review draws upon author findings and also uses primary evidence from each paper (i.e. synthesizing author‐extracted themes, quotes, etc.) to construct an interpretation of the wider qualitative evidence base. Whilst it is not possible to determine the extent of confirmability 90 for each individual study, every effort was made to ensure our findings were not influenced by researcher bias, motivation or interest. Moreover, similar themes, barriers and facilitators were identified across the wide range of included studies. This implies that the perceptions around activity behaviours of those (women) caring for young children are relatively dependable (i.e. consistent) and transferable (i.e. stable across contexts such as location and time) 90.

## Conclusions

This synthesis of qualitative evidence of barriers and facilitators to activity behaviours in preschool‐aged children identified seven broad themes, highlighting the perceived influences of the child, the home, out‐of‐home childcare, parent–childcare provider interactions, environmental factors, safety and weather. Similarities were apparent between previously explored determinants from quantitative studies, and the barriers and facilitators to activity behaviours identified here, particularly at the interpersonal and organizational levels. Thoughts and beliefs of men caring for young children, those from developing countries and about barriers and facilitators in the environmental and policy domains are currently lacking. Nevertheless, this qualitative synthesis compliments previous research and provides new as‐yet unexplored targets for intervention (e.g. the parent–childcare provider interaction, safety and weather). By focusing on factors that those caring for children 0–6 years of age believe to be important, it may be possible to enhance intervention tailoring, and ultimately effect greater positive change on young children's activity behaviours.

## Contributions

This review is part of a larger project to explore the determinants of obesogenic behaviours in children aged 0–6 years, conducted as part of the School of Public Health Research by researchers at Cambridge and Durham Universities. Kathryn Hesketh (K. H.) led the physical activity reviews. K. H. screened one‐third of the initial titles, conducted the updated search (specific to physical activity and sedentary behaviour in 2016), screened all new references and read all identified texts in full to determine which were eligible for inclusion. K. H. extracted and synthesized the data, and E. V. S. double screened 15% of the included papers for fidelity. K. H. and E. V. S. interpreted the findings of this review and developed the theoretical framework with the help of R. L. K. H. drafted the manuscript, and all authors reviewed and approved the final manuscript.

## Funding

This is an outline of independent research funded by the National Institute of Health Research, School for Public Health Research (NIHR SPHR). The views expressed are those of the author(s) and not necessarily those of the National Health Service, the NIHR or the Department of Health. The NIHR SPHR is a partnership between the Universities of Sheffield, Bristol, Cambridge, UCL; The London School for Hygiene and Tropical Medicine; The Peninsula College of Medicine and Dentistry; the LiLaC collaboration between the Universities of Liverpool and Lancaster and Fuse; The Centre for Translational Research in Public Health; and a collaboration between Newcastle, Durham, Northumbria, Sunderland and Teesside Universities.

K. H. is funded by the Wellcome Trust (107337/Z/15/Z). This work was also supported by the Medical Research Council (unit programme number MC_UU_12015/7) and undertaken under the auspices of the Centre for Diet and Activity Research, a UKCRC Public Health Research Centre of Excellence, which is funded by the British Heart Foundation, Cancer Research UK, Economic and Social Research Council, Medical Research Council, the National Institute for Health Research, and the Wellcome Trust (RES‐590‐28‐0002).

## Conflict of interest statement

No conflict of interest was declared.

## Supporting information

Table S1. Search strategy for full review and physical activity‐specific update
Table S2. Quality assessment criteria and operationalisation
