# Supplementary material for: Barriers and facilitators to young children's physical activity and sedentary behaviour: a systematic review and synthesis of qualitative literature
Source: Obes Rev. 2017 Jun 6;18(9):987–1017. doi: 10.1111/obr.12562 (PMC5575514; doi:10.1111/obr.12562)
Supplement: Supplementary file 1 — Table S1. Search strategy for full review and physical activity‐specific update Table S2. Quality assessment criteria and operationalisation [file OBR-18-987-s001.docx]

Supplementary Table S1: Search strategy for full review and physical activity-specific update

|  | | |
| --- | --- | --- |
| 1 | (Determin*4 or correlates or factors or predict*3 or associate*3 or interaction or influence*1 or temperament or beliefs or attitudes or knowledge or perceptions or views or intentions or facilitators or barriers or experiences or prevent*3 or reduc*5 or increas*3 or promot*3 or education or curriculum or program*3 or polic*3 or media or campaign or review or intervention*1 or initiative*1 or strategy*3 or evaluation or trial).mp. [mp=title, abstract, original title, name of substance word, subject heading word, protocol supplementary concept, rare disease supplementary concept, unique identifier] |  |
| 2 | (Infant* or Toddler* or Preschool* or Nurser*).mp. [mp=title, abstract, original title, name of substance word, subject heading word, protocol supplementary concept, rare disease supplementary concept, unique identifier] |  |
| 3^a^ | ((Fruit*1 or Vegetable*1 or juice or sugar sweetened beverage*1 or fizzy drinks or soft drinks or junk food or fast food or processed food or unhealthy food or takeaway food or non-core food or energy dense food or high fat food or fatty food or nutrient poor food or unhealthy diet or healthy eating or portion size or empty calories or confectionery or sweet*1 or dessert*1 or chocolate*1 or cake*1 or biscuit*1 or burger*1 or chip*1 or crisp*1 or snack*1 or breakfast or lunch or dinner or obes*6 or overweight).mp. [mp=title, abstract, original title, name of substance word, subject heading word, protocol supplementary concept, rare disease supplementary concept, unique identifier] |  |
| 4 | (physical activ*5 or inactiv*3 or exercise*1 or outdoor or TV or Television or Tele or sedentary or (screen adj time)).mp. [mp=title, abstract, original title, name of substance word, subject heading word, protocol supplementary concept, rare disease supplementary concept, unique identifier] |  |
| 5 | 1 AND 2 AND 3 AND 4 |  |
| 6 | 5 not (cerebral palsy or asthma or cystic fibrosis or autism).mp. [mp=title, abstract, original title, name of substance word, subject heading word, protocol supplementary concept, rare disease supplementary concept, unique identifier] |  |
| ^a^ | Search terms omitted in updated search conducted in July 2016 |  |

| **Assessment Criteria** | **Operationalisation** |
| --- | --- |
| - Research/ methods justified - Appropriateness of approach - Methods clearly described - Sufficiency of evidence | Research questions clearly stated: 1  Qualitative approach clearly justified: 1  Study context clearly described: 1  Sampling strategy appropriate for the research question: 1  Data collection method appropriate: 1  Approach appropriate for the research question: 1  Analysis appropriate for the research question: 1  Sampling method clearly described: 1  Role of the researcher clearly described: 1  Method of data collection clearly described:1  Method of analysis clearly described: 1  Conclusions supported by sufficient evidence: 1 |
| **Total possible score** | **12** |

Supplementary Table S2: Quality assessment criteria and operationalisation
